# Primary diffuse leptomeningeal glioblastoma: a case report and literature review

**DOI:** 10.1007/s11060-024-04908-8

**Published:** 2024-12-12

**Authors:** Mark Willy L. Mondia, Rebekka E. Hooks, Georgios A. Maragkos, Vanessa L. Smith, Matthew R. McCord, Joseph H. Donahue, Eli S. Williams, M. Beatriz Lopes, David Schiff, Ashok R. Asthagiri

**Affiliations:** 1 Division of Neuro-Oncology, Department of Neurology, University of Virginia, 1300, Jefferson Park Avenue West Complex Room 6225, Charlottesville, 22908 VA USA; 2 Department of Neurosurgery, University of Virginia, Charlottesville, VA USA; 3 Department of Pathology, University of Virginia, Charlottesville, VA USA; 4 Department of Radiology and Medical Imaging, University of Virginia, Charlottesville, VA USA

**Keywords:** Glioblastoma, GBM, Leptomeningeal disease, LMD, Leptomeningeal spread, LMS

## Abstract

**Purpose:**

Glioblastoma (GBM) that presents as leptomeningeal disease (LMD) is extremely rare and fatal. Limited data are available regarding incidence, clinical presentation, and management. Prognosis is poor and no treatment is known to improve survival.

**Methods and results:**

We present a case report of a 72-year-old female who presented with depressed sensorium, ataxia, and myelopathy. Magnetic resonance imaging (MRI) showed diffuse supratentorial and spinal LMD. There was an absence of any detectable and distinct intraparenchymal lesion on neuroaxis imaging. Biopsy of the Sylvian fissure nodule revealed GBM. Steroid therapy was ineffective for symptom relief. She opted for palliative care and expired shortly after diagnosis.

**Conclusion:**

To our knowledge, this is the first reported case of GBM presenting exclusively as LMD without a primary lesion. If systemic imaging techniques do not provide a biopsy target and cerebrospinal fluid (CSF) studies are non-diagnostic, tissue diagnosis from leptomeningeal biopsy is recommended. Palliative chemoradiation or best supportive care are reasonable treatment options.

**Supplementary Information:**

The online version contains supplementary material available at 10.1007/s11060-024-04908-8.

## Introduction

Glioblastoma (GBM) is the most common malignant primary brain tumor in adults with an incidence of 3 per 100,000 person years [1]. The most common presentation is with a dominant parenchymal lesion exhibiting contrast enhancement and perilesional T2/FLAIR signal change. Standard of care therapy remains maximal safe resection followed by radiotherapy with concomitant and adjuvant temozolomide [2].

Leptomeningeal disease (LMD) secondary to leptomeningeal spread (LMS) is a rare and severe complication of GBM resulting from tumor cells disseminating from the brain parenchyma to the meninges and cerebrospinal fluid (CSF) [3]. Incidence may be above the initially reported 2–4% and as high as 15–25% and survival is estimated between 0.2 and 9.7 months [3–8]. The majority (90%) of LMS is termed “secondary LMS”, is detected during recurrence, and usually occurs within the first 2 years of the disease course. LMS during initial diagnosis of GBM is termed “primary LMS” and is extremely rare [9].

Of particular note, there is no published data on GBM manifesting exclusively as LMD without dissemination from a well-defined dominant mass of the brain parenchyma. We report a case of a 72-year-old woman who was diagnosed with GBM presenting as LMD without a primary parenchymal lesion.

## Case report

**Fig. 1:**
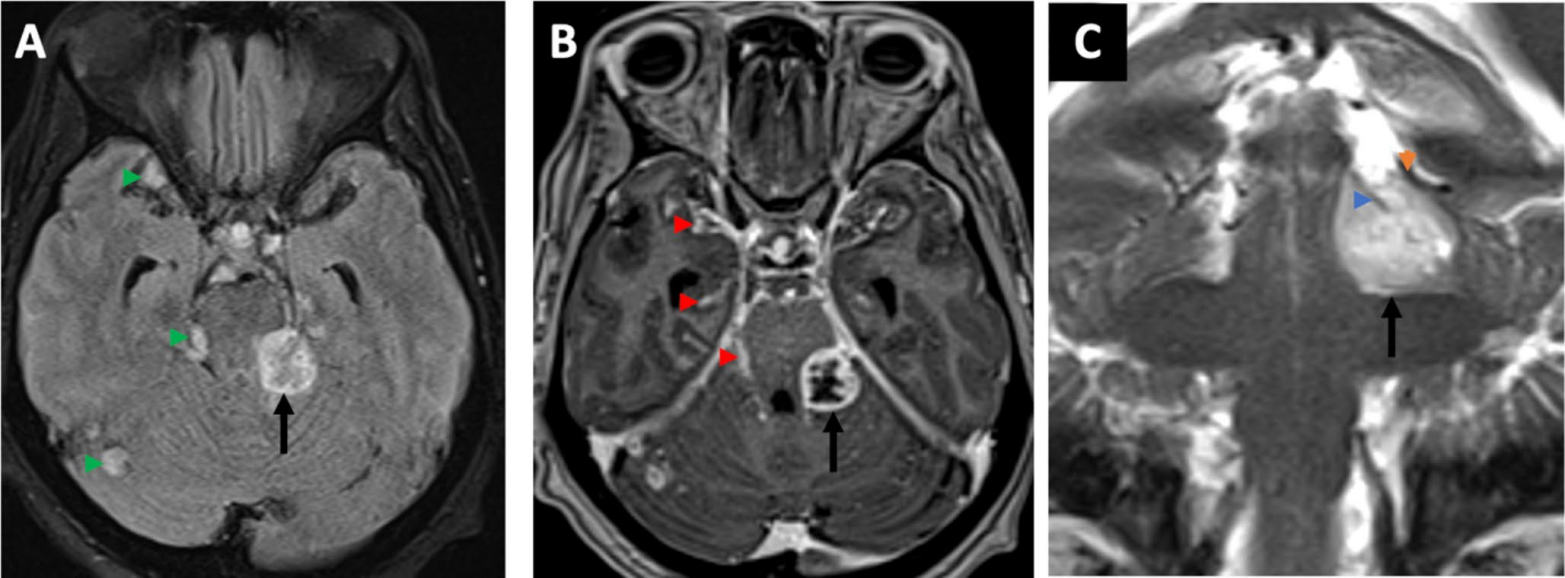
MRI brain. Axial FLAIR (**A**), Axial postcontrast T1-weighted MPRAGE (**B**), Coronal T2-weighted imaging (**C**). Nodular leptomeningeal tumor deposits are appreciable on FLAIR images as discrete foci of hyperintense signal abnormality within and around the basilar cisterns (green arrowheads in **A**). The largest tumor deposit (black arrow in **A-C**) demonstrated central necrosis on post-contrast imaging (black arrow in **B**) with corresponding hyperperfusion on cerebral blood volume map (not shown). More subtle diffusely disseminated smooth leptomeningeal enhancement is also noted on post-contrast imaging (red arrowheads in **B**). The extra-axial location of the largest tumor deposit compressing the left dorsal pons (black arrow in **A-C**) is better demonstrated on coronal T2-weighted images with lesional abutment of the tentorial leaflet (orange arrowhead in **C**) and encasement of the superior cerebellar artery flow void (blue arrowhead in **C**). *Abbreviations: MRI: magnetic resonance imaging*,* FLAIR: fluid-attenuated inversion recover*,* MPRAGE: Magnetization Prepared - RApid Gradient Echo*

**Fig. 2:**
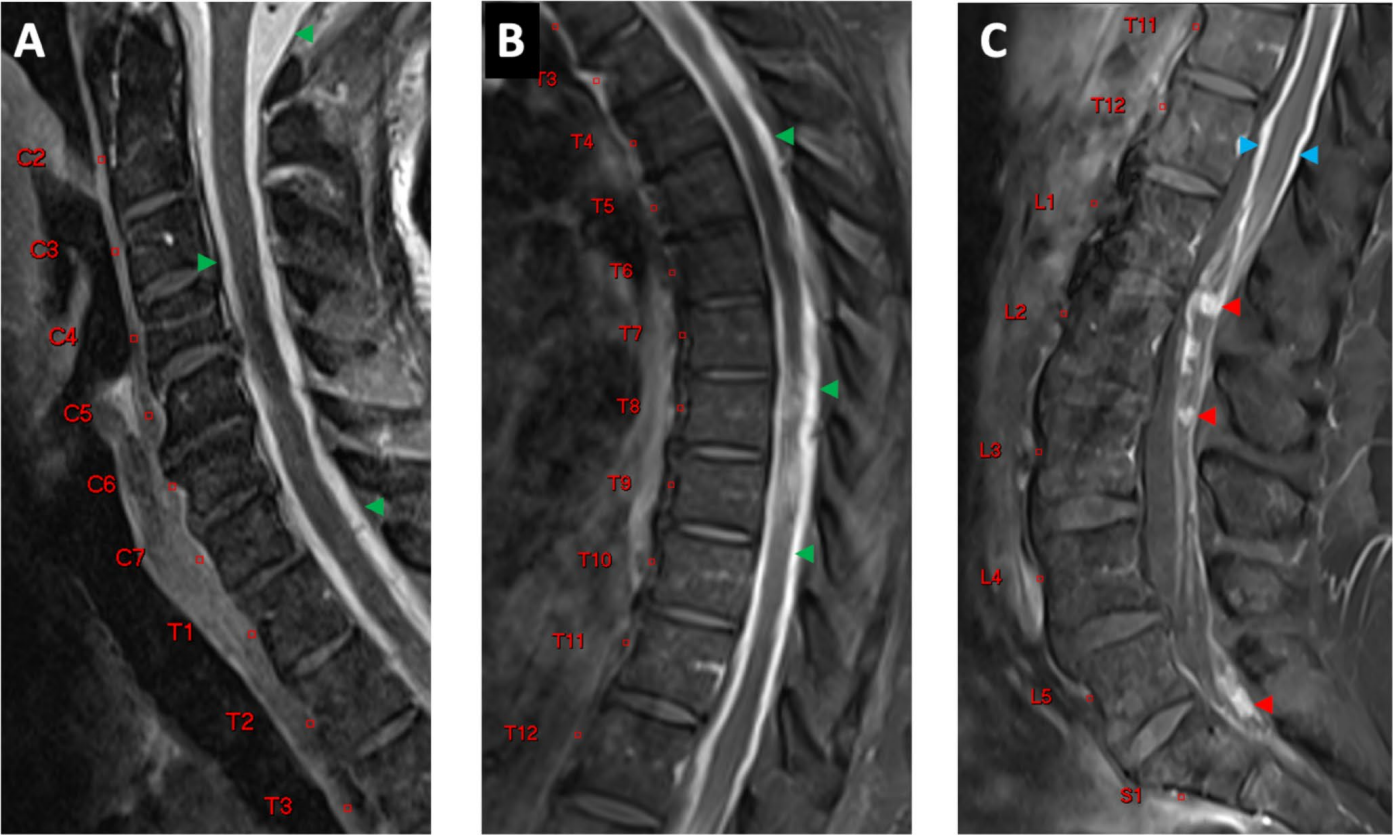
MRI Spine. Sagittal T1-weighted postcontrast MRI of the Cervical (**A**), Thoracic (**B**), and Lumbar (**C**) spine. Sagittal T1-weighted postcontrast imaging of the cervical and thoracic spine demonstrates diffusely hyperintense subarachnoid spaces within the foramen magnum and intraspinal compartments (green arrowheads in **A** and **B**), which reflects a combination of extensive leptomeningeal tumor deposits and associated leakage of gadolinium contrast agent into the subarachnoid space. Sagittal t1-weighted postcontrast imaging of the lumbar spine demonstrates both smooth (blue arrowheads in **C**) and nodular (red arrowheads in **C**) leptomeningeal enhancement of the conus surface and cauda equina, respectively

**Fig. 3:**
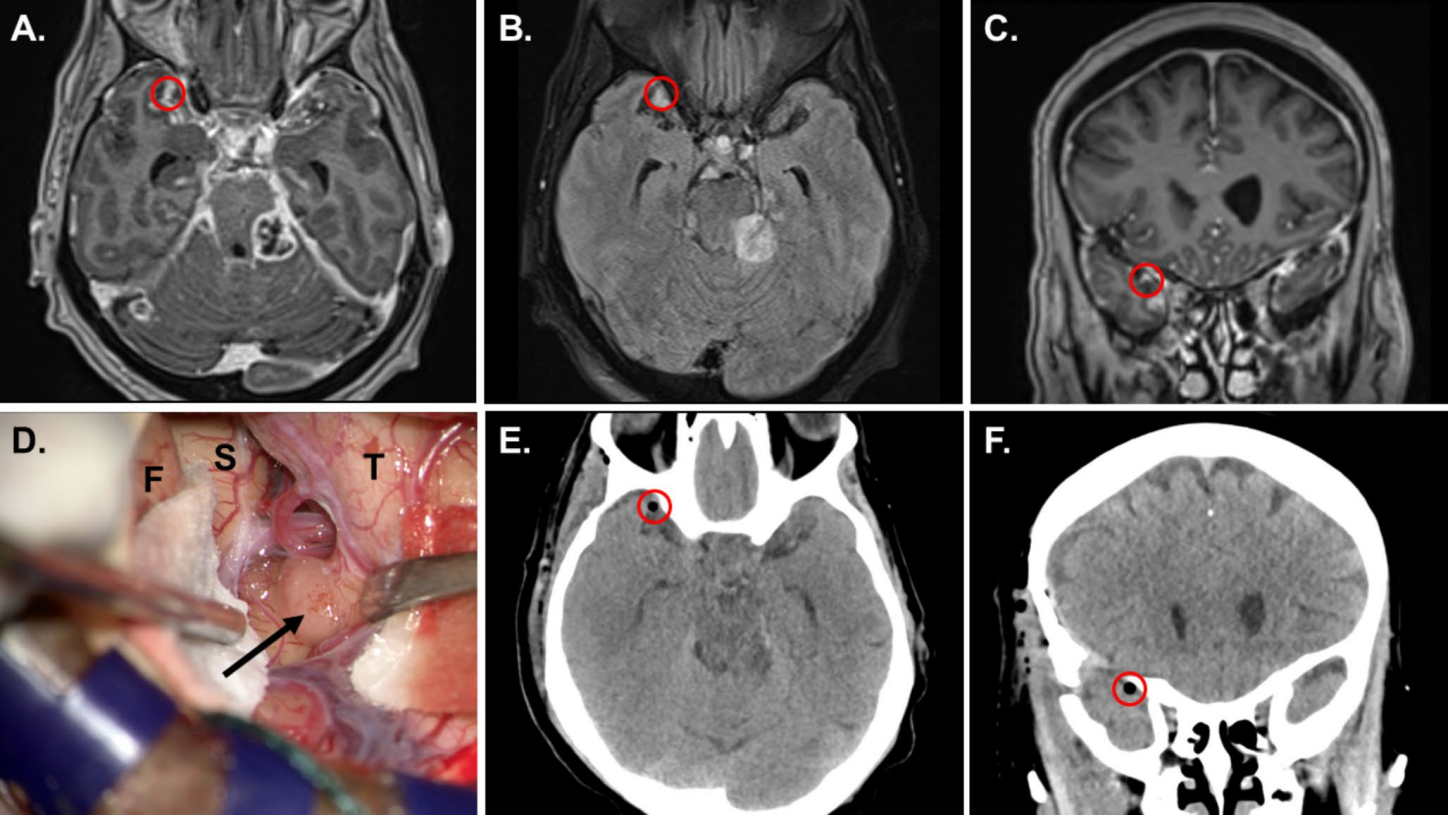
Axial T1 post-contrast (**A**), axial FLAIR (**B**), and coronal T1 post-contrast MRI sequences (**C**), demonstrating the target lesion (red circle), as an extra-axial, contrast-enhancing, FLAIR-hyperintense nodule. Several additional extra-axial, contrast-enhancing lesions are also evident. (**D**) Intraoperative photograph of the target lesion (arrow) as viewed through the operative microscope. The lesion resides within the arachnoid meningeal layer, within the Sylvian fissure (S), between the frontal (F) and temporal (T) lobes, which are separated and retracted. Postoperative axial (**E**), and coronal (**F**) CT scans, demonstrating a small area of pneumocephalus (red circle), where the biopsy was performed and marked with gelfoam, corresponding well with the preoperative target. *Abbreviations: FLAIR: fluid-attenuated inversion recovery*,* CT: computed tomography*

A 72-year-old female presented with a 4-week history of progressive imbalance resulting to multiple falls. She did not experience any headaches, vision changes, seizures, focal weakness, or numbness. On review of symptoms, she reported intermittent urinary retention. While at work, she had an episode of acute confusion, dizziness, and ataxia that resulted in a major fall leading to her hospitalization. During her hospital course, she developed progressive bilateral lower extremity weakness and intermittent episodes of confusion. On examination, she had waxing-waning sensorium and at best was only oriented to herself. Nonetheless, she was able to follow commands on prodding. She had poor recall and attention, but had no aphasia, dysnomia, or dysarthria. She did not have any cranial nerve deficits. Her upper extremities did not exhibit any abnormalities in motor, sensory, and cerebellar testing. She had signs of incomplete myelopathy specifically preferential weakness of her bilateral lower extremities (2/5 by manual muscle testing) with bilateral hyporeflexia and impaired proprioception. A reliable spinal sensory level was difficult to confirm due to depressed sensorium. She had no history of incontinence and had good rectal tone, however, she necessitated urinary catheterization. Signs for meningeal irritation were absent. Gait testing was not possible due to her paraparesis. Central nervous system (CNS) MR-imaging of the brain and whole spine were done due to progressive myelopathy in the setting of acute encephalopathy. These revealed extensive intracranial (Fig. 1) and spinal leptomeningeal enhancement (Fig. 2). CSF studies were obtained to attempt a minimally invasive histopathologic diagnosis. Additionally, this may aid in narrowing down the differential diagnosis (i.e., to rule out an infectious or demyelinating process). Lumbar puncture opening pressure was within normal limits (5 mm H_2_O). CSF was acellular but had grossly elevated protein of 3,659 mg/dL, and increased lactate of 3.8 mmol/L. However, CSF cytology and flow cytometry were inconclusive. CT scans of the chest, abdomen, and pelvis did not reveal any infectious, inflammatory or neoplastic processes outside the CNS. She was started on dexamethasone 8 mg/day after the lumbar puncture and prior to surgery due to the degree of her neurologic dysfunction with a Karnofsky Performance Score (KPS) of 30. She had minimal improvement of symptoms despite steroid therapy. She then underwent right pterional craniotomy for identification and open biopsy of a small Sylvian fissure region nodule located between arachnoid and pia (Fig. 3).

**Fig. 4:**
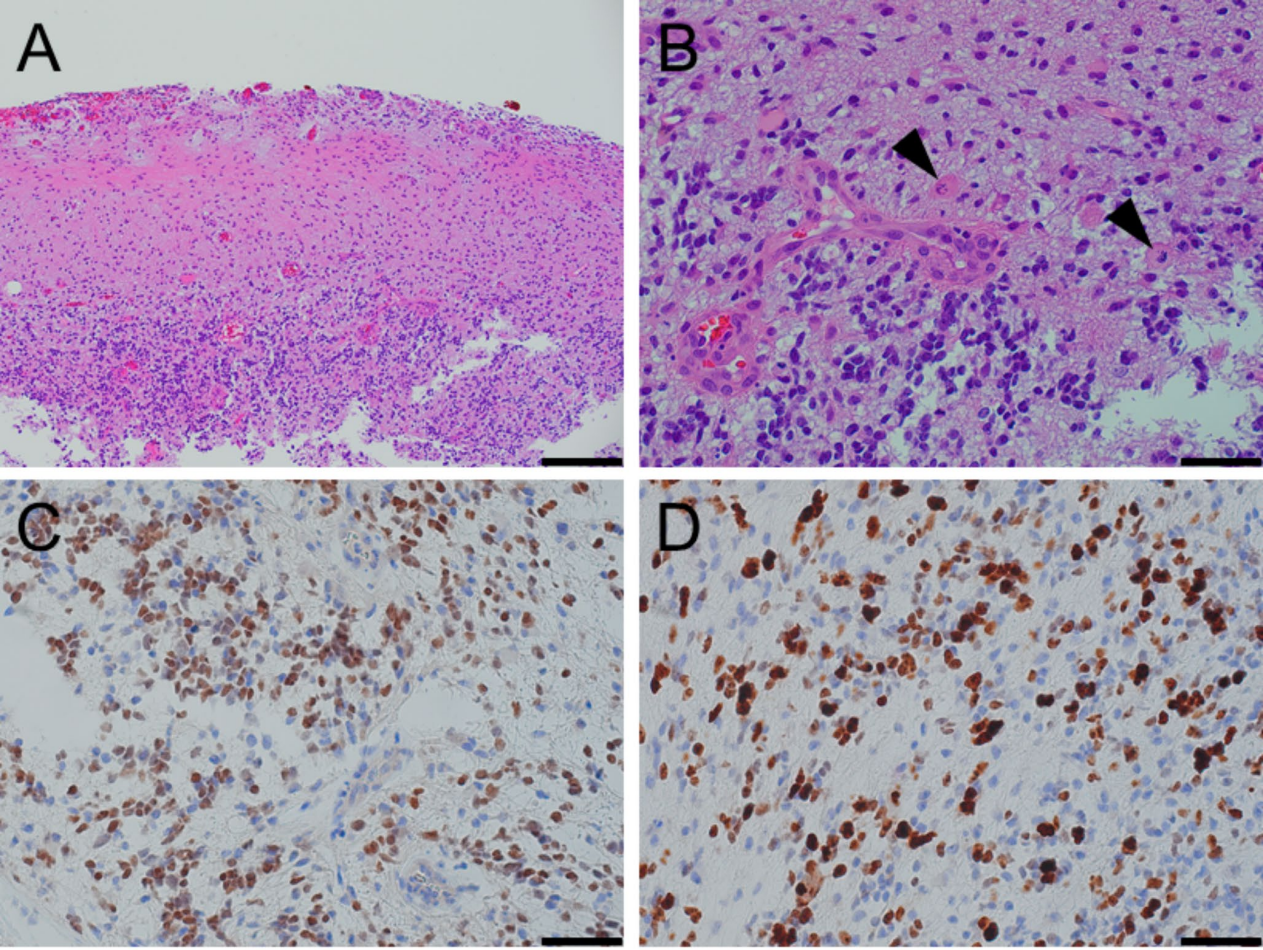
Tumor histopathology and immunophenotype. (**A**) Low-power view of H&E-stained section shows superficial cortex with a diffusely infiltrating glial neoplasm. (**B**) High-power view of H&E-stained section shows astrocytic nuclear morphology, mitotic figures (arrowheads), and microvascular proliferation. (**C**) High-power view of nuclear positivity for Olig-2 protein in many tumor cells. (**D**) High-power view of elevated Ki-67 proliferation index in tumor. Scale bar = 200µM in panel **A**, and 50µM in panels **B-D**

Pathology confirmed the diagnosis of glioblastoma, IDH-wildtype, CNS WHO grade 4. The tumor involved the superficial cortex and leptomeninges, with a diffusely infiltrative growth pattern (Fig. 4A). Tumor cells showed astrocytic morphology. Increased mitotic activity and microvascular proliferation were present (Fig. 4B). Tumor cells were positive for Olig-2 (Fig. 4C), and Ki-67 proliferation index was markedly elevated (Fig. 4D). PGDx next-generation sequencing showed that the tumor was wild-type for IDH1/2 and histone mutations, and had a pathogenic TERT promoter mutation. All detected sequence variants are listed in Supplementary Table S1. Pyrosequencing showed that the O^6^-methylguanine-DNA methyltransferase (MGMT) promoter was hypermethylated (Supplementary Table S2).

The patient was clinically stable post-biopsy, but her functional status did not improve. After a goals of care discussion considering the widely disseminated nature of her disease and her low performance status, the patient and her family opted for palliative care. She has been on dexamethasone 8 mg/day for total of 18 days at this point. She was discharged on dexamethasone 4 mg/day with a planned taper schedule. She passed away 4 days after being discharged.

In summary, this 72-year-old female was admitted for chronic progressive ataxia and intermittent urinary retention punctuated by an acute deterioration presenting with decreased sensorium and worsening paraparesis. Her examination was significant for mild to moderate encephalopathy and incomplete myelopathy affecting the bilateral lower extremities. MRI of the brain and spinal cord revealed diffuse leptomeningeal disease with nodular enhancements concentrated in the basal cisterns without intraparenchymal lesions. CSF analysis was performed to rule out non-neoplastic etiologies and attempt to achieve cytologic diagnosis. Although there was significant hyperproteinosis, the CSF cytology was non-diagnostic. Systemic malignancy screening utilizing CT scans was also negative. Hence, tissue biopsy was necessary and confirmed the histopathologic and molecular diagnosis of GBM. She did not improve with prolonged oral steroid therapy and eventually succumbed to the disease.

## Discussion

Leptomeningeal involvement in GBM is rare and was first documented in 1981 [10]. The largest cohort was reported in 1990, where 2% of 600 patients with supratentorial GBMs had symptomatic LMS [11]. Most reported cases are secondary LMS [9]. To date, only 15 cases have been published documenting primary LMS [9]. Particularly, primary LMS without a focal intraparenchymal or spinal lesion at initial diagnosis of GBM has not been reported. An unpublished abstract presented at the 2024 annual meeting of the American Association of Neuropathologists reported 5 cases of adult primary diffuse leptomeningeal gliomatosis (PDLG) with three of these patients having FLAIR abnormalities in the brain parenchyma. The leptomeningeal biopsies showed molecular features of GBM, but pathology showed astrocytic gliomas with no definite microvascular proliferation or necrosis [12]. Less than 150 cases of PDLG in high grade gliomas were identified prior to using molecular markers to diagnose GBM [13, 14]. Thus, it is still uncertain whether PDLG is molecularly equivalent to GBM with leptomeningeal involvement [15]. Notably, a case of GBM with H2K27M mutation presenting as LMD was reported to have no mass lesion, but had a small area of cerebellar intraparenchymal enhancement [16]. To the best of our knowledge, our case is the first report on primary diffuse leptomeningeal GBM– that is GBM presenting as disseminated leptomeningeal infiltration without a clear primary parenchymal lesion. As seen in Fig. 1, the largest lesion abutting the left dorsal midbrain is extra-axial, and there exist several other leptomeningeal deposits of varying size, but there were no intraparenchymal sites of pathology. We feel this further strengthens our point regarding this case being primary leptomeningeal GBM.

Glioblastoma cells usually migrate from the initial tumor site along the vasculature to reach the subarachnoid space and disseminate along the CSF route [3]. Exclusive leptomeningeal affectation at diagnosis may represent occult tumor cells that metastasized early. A microscopic primary intraparenchymal lesion undetectable by neuroimaging may also be a possibility.

Clinical presentation varies and could correlate with anatomical involvement, but can range from being asymptomatic to symptomatic severities such as impending herniation [7, 17]. Acute worsening of symptoms is not unusual [18]. Sensorial and behavioral changes are commonly noted in the elderly [19]. Our patient presented primarily with imbalance, cognitive issues, and eventually myelopathy. Topographic localization was suspicious for widespread cortical and spinal lesions.

Risk factors that predispose to LMS in GBM include age (35–45 years old), location, male gender, increased progression free survival, and tumor volume [4, 5, 11, 19]. Infratentorial location, spatial proximity to ventricles, and environment of the subventricular zone have been reported with higher frequency of LMS [6, 20, 21]. Ventricular opening during surgery, repeated surgeries, and persistence of preoperative leptomeningeal enhancement were correlated with higher LMS incidence [22–24]. However, prophylactic radiation was not beneficial [25].

Radiologic screening of the neuroaxis is recommended for GBM with suspected LMS including cranial nerve deficits, progressive headaches, and spinal cord symptoms [26]. Brain MRI findings typically noted include nodular, pial, or nerve root enhancement [27]. Spinal involvement is reported in the thoracic (52%), lumbar (41%), and cervical (31%) levels [28]. The patient’s brain and spine MRI imaging exhibited all of the forementioned typical findings. The absence of a primary discrete intraparenchymal lesion discernible by neuroimaging is a unique feature of this case.

Multicentricity increases targets for tissue diagnosis, but this may also present unique concerns regarding the ideal surgical approach. Stereotactic needle biopsy in the brainstem is certainly indicated in situations where the brainstem is the only location of pathology, such as in brainstem gliomas (e.g. diffuse intrinsic pontine gliomas). However, stereotactic needle biopsy of brainstem targets has long been associated with significant risk and morbidity, including uncontrollable bleeding into the subarachnoid cisterns or consequential neurologic deficits (e.g., dysconjugate gaze, swallowing and breathing disorders, or quadriplegia) [29–32]. In our case, when weighing the options of bleeding into the subarachnoid cisterns versus those associated with a small and restricted pterional approach, the latter appeared to be the safer technical approach. The patient experienced no additional morbidity as a result of surgical exposure and biopsy, and this does - in hindsight - still appear to be the appropriate approach for diagnosis in this complicated situation.

Malignant cells are seen in CSF in 25–45% after the first assay, but can reach to 93% with more than 3 lumbar punctures [33]. However, the positivity can still range from 4 to 75% in radiographically confirmed leptomeningeal affection; making CSF analysis ancillary but not necessary for diagnosis [34]. A retrospective study of 168 glioma patients with LMD at recurrence reported 25% (11 out of the 45 patients who underwent lumbar puncture) CSF positivity [7]. It is therefore not surprising that CSF cytology will be negative even in the presence of heavy disease burden as was the case in our patient. Important to note that the patient’s CSF protein level (3, 659 mg/dL ) is substantially beyond the expected increase reported by previous studies (50–100 mg/dL) [18, 35, 36]. Interestingly, despite the extremely elevated CSF protein and strategic location of nodular leptomeningeal deposits to obstruct CSF flow, the patient did not have severe signs and symptoms of increased intracranial pressure to necessitate CSF diversion.

The tumor showed typical morphology and immunophenotype of IDH-wildtype glioblastoma, with astrocytic cytology, diffusely infiltrative growth, brisk mitotic activity with elevated Ki-67 proliferation index, and microvascular proliferation. Molecular features predicting high risk of leptomeningeal dissemination have not been extensively studied in adult-type diffuse gliomas. Extant data suggests that IDH-wildtype status and absence of MGMT promoter hypermethylation are associated with increased risk of LMD [37]. It is interesting that this case had such marked hypermethylation of the MGMT promoter (between 87 and 93% on the CpGs tested). In addition to TERT promoter mutation, this case had likely pathogenic mutations in CDKN2C, PIK3CA, and PTEN. PIK3CA and PTEN mutations have been commonly reported in glioblastoma, though the PIK3CA and PTEN mutations are typically mutually exclusive. The potential role of these mutations in development of LMD in this case is not entirely clear.

There is no consensus on treatment for this end-stage GBM disease state [38]. Surgery is reserved to establish histopathologic diagnosis and for symptom benefit, but does not improve survival [39]. Palliative radiotherapy combined with multiple chemotherapeutic agents (temozolomide, lomustine, procarbazine, thiotepa, cytarabine, methotrexate, topotecan, irinotecan, etoposide and platinum-based agents) have been used mainly for symptom control [40–42]. Targeted therapy (bevacizumab) and immunotherapy have not demonstrated meaningful clinical benefit [43, 44]. Clinical trials looked into intrathecal topotecan, methotrexate, and cytarabine, but did not show improvement in survival [45, 46]. A recent review supports best supportive care by a multidisciplinary team as a suitable approach [3].

Our report has limited generalizability and does not generate epidemiological data. But it is important to document clinicopathologic, radiologic, and diagnostic characteristics of this very rare presentation of GBM to broaden the understanding of this aggressive and fatal disease. There can be presence of researcher, publication, and recall biases that are inherent to case reports. Lastly, the authors may be prone to mischaracterize, overinterpret or misinterpret certain aspects of the case. We recognize that underreporting and rapid demise in similar cases may be factors contributing to the scarcity of published cases, which may undermine the novelty of our case report.

In conclusion, the possibility of a high-grade primary diffuse leptomeningeal glioma like GBM, although extremely rare, should be considered in any case of LMD. Other primary CNS tumors such as glioneuronal tumor, astrocytoma, oligdendroglioma, medulloblastoma, ependymoma, and pineoblastoma can also affect the leptomeninges [47]. More common differential diagnoses such as metastatic carcinoma, CNS lymphoma, infectious etiologies, and demyelinating disease should be ruled out by performing a lumbar puncture and screening for systemic malignancy. Nuclear imaging may be pursued in cases with high-risk for metastatic disease to identify a primary lesion prior to more invasive CNS tissue diagnosis. Unfortunately, treatment remains limited with dismal prognosis once the leptomeninges are involved in the disease process. There is an unmet need for more robust prospective studies to elucidate the epidemiology, pathophysiology, molecular mechanism, natural progression, and management of this fatal GBM complication.

## Electronic supplementary material

Below is the link to the electronic supplementary material.

Supplementary Material 1

## Data Availability

No datasets were generated or analysed during the current study.
