# Supplementary material for: Primary diffuse leptomeningeal glioblastoma: a case report and literature review
Source: J Neurooncol. 2024 Dec 12;172(1):265–72. doi: 10.1007/s11060-024-04908-8 (PMC11832630; doi:10.1007/s11060-024-04908-8)
Supplement: Supplementary file 1 — Supplementary Material 1 [file 11060_2024_4908_MOESM1_ESM.docx]

| **gene** | **status** | **alteration** | **Pathogenicity** | **VAF (%)** |
| --- | --- | --- | --- | --- |
| TERT | mutant | promoter | Pathogenic | 47.3 |
| CCNE1 | mutant | Q314* | VUS | 51.4 |
| CDKN2C | mutant | E151* | Likely Pathogenic | 16.5 |
| FANCD2 | mutant | H1070L | VUS | 50.2 |
| FLT4 | mutant | R1127Q | VUS | 41.8 |
| MED12 | mutant | L836F | VUS | 44.9 |
| NF1 | mutant | A617T | VUS | 54.8 |
| NKX3-1 | mutant | P7T | VUS | 43.3 |
| NOTCH4 | mutant | L1638Rfs*68 | VUS | 54 |
| PIK3CA | mutant | R38H | Likely Pathogenic | 42.1 |
| PRKDC | mutant | Y1571C | VUS | 45.9 |
| PTEN | mutant | V53A | Likely Pathogenic | 88.8 |
| RANBP2 | mutant | F1540L | VUS | 46.5 |
| TLR7 | mutant | F873V | VUS | 48.6 |
| TLR8 | mutant | F834S | VUS | 46.1 |
| IDH1/2 | wild-type | N/A |  | N/A |
| H3F3A | wild-type | N/A |  | N/A |

Supplementary Table S1: Sequence variants

| **MGMT exon 1 site** | **percent methylation** |
| --- | --- |
| site 1 | 87% |
| site 2 | 88% |
| site 3 | 93% |
| site 4 | 93% |

Supplementary Table S2: MGMT pyrosequencing
